# Work on the cutting edge: metallographic investigation of Late Bronze Age tools in southeastern Lower Austria

**DOI:** 10.1007/s12520-021-01378-1

**Published:** 2021-07-02

**Authors:** Marianne Mödlinger, Peter Trebsche

**Affiliations:** 1 Dipartimento di Chimica e Chimica Industriale (DCCI), Università degli Studi di Genova, Genoa, Italy; 2 Institut für Archäologien, Leopold-Franzens-Universität Innsbruck, 6020 Innsbruck, Austria

**Keywords:** Late Bronze Age, Eastern Alps, Austria, Mining site, Metallographic analysis, XRF analyses, Production of copper alloy objects, Chaîne opératoire

## Abstract

This paper analyses 20 Late Bronze Age (ca 1080–800 BC) copper alloy objects to discern their manufacture and the skills of local craftsmen. Several tools and jewellery were studied that originated from a bronze workshop located immediately next to the Prigglitz-Gasteil copper ore mining site and several contemporaneous sites in the surrounding area. The samples were studied with optical microscopy (microstructurally), and SEM-EDXS and XRF (chemical analyses). Our analyses are part of a larger study and suggest that the Prigglitz region’s bronze production was not standardized. Particular alloys do not seem to have been chosen for object types or due to their intended use-function. Notably, approximately 20% of the objects contain unalloyed copper inclusions, which are most likely a result of the incomplete mixing of scrap metals and alloys during their production.

## Introduction

Numerous bronze casting workshops have been found that belong to the Middle Danubian Urnfield Culture dating to the Late Bronze Age (ca 1300–800 BC) in eastern Austria, southern Moravia, southwestern Slovakia, and western Hungary (Lochner, 2013). Evidence of archaeological and metallurgical remains in these regions (e.g. casting moulds and debris and semi-finished products) shows that metalworking was concentrated in central hillforts at sites such as Szentvid near Velem (comitate of Vas), and Gór-Kápolnadomb (comitate of Vas) and Várvölgy (comitate of Zala) in western Hungary (Ilon, 1992, 1996, 2018; Müller, 2006; Czajlik, 2014). In adjacent Lower Austria, metal workshops are assumed to have existed at the hillforts Schanzberg near Thunau am Kamp (Lochner, 2004, 2017), ‘Gelände’ near Grünbach am Schneeberg (Mühlhofer, 1952; Trebsche et al., 2019, and Rauheneck near Baden (Calliano, 1894, 90). The raw metal that supplied these workshops is disputed; however recent archaeometallurgical investigations of copper alloys from the region suggest that there were likely several ore mining sites that supplied Late Bronze Age metalworkers in the Middle Danube region (Czajlik, 2013; Zachar and Salaš, 2018, 2019; Mödlinger and Trebsche, 2020).

During recent excavations at the Prigglitz-Gasteil site, in the Neunkirchen district, an extraordinary Late Bronze Age casting workshop was discovered. The site is not located at a hillfort but immediately next to a contemporaneously dated copper ore mine at the slopes of the Gahns mountain in the Schneeberg-Rax region of southeastern Lower Austria. Excavations at the site from 2010 to 2014, and subsequent geophysical surveys and core drillings from 2017 to 2018 (Trebsche, 2013, 2015b, 2015a; Trebsche and Pucher, 2013; Haubner, et al., 2019), have shown that copper ore, mainly chalcopyrite and pyrite mineralization, were extracted from opencast mines at the site during the Late Urnfield Period (Ha B, ca 1080 to 800 BC). The dwellings and workshops of Late Bronze Age miners and craftsmen at the site were constructed on artificial terraces cut into the heaps of mining debris. During the excavations, two terrain terraces, T3 and T4, were investigated in detail, yielding evidence for bronze casting activities. The evidence consists of numerous casting drops and fine platy slags that predominantly belong to three occupation phases: T3-10, T3-08F, and T3-08A.

On the upper terrain terrace of T3, which according to a series of radiocarbon dates, was in use from the end of the tenth century BC to the end of the ninth century BC (Trebsche, 2015b; Trebsche, in preparation), only casting waste and fragments of casting tools were found so that the spectrum of production is unknown. However, four finds from the  site are important as they indicate the production of at least three categories of bronze objects: first, one fragment of a sandstone casting mould for a knife with a tang hilt (*Griffdornmesser*, probably type Baumgarten after Říhovský, 1972, 64–71; Trebsche, 2015b, 49, Fig. 2/7); second, one sandstone casting cone for the production of a socketed axe; third, a bronze casting cone that fits into the socket of small arrowheads, indicating on-site weapon production; fourth, a casting sprue that cannot be precisely attributed to an artefact type but is the size appropriate for casting an object like a knife, razor, or sickle (Trebsche and Pucher, 2013, 127–128, Fig. 14/2). Hence, the workshop at Prigglitz-Gasteil seems to have produced a range of artefacts that indisputably included arrowheads, knives (*Griffdornmesser)*, and socketed axes.

No heavy tools, such as hammers, axes and pickaxes, or weapons like swords, were found at the site; only small objects such as rings, belt clips, double-pointed tools, two completely preserved knives, and two dress pins were discovered during the excavations. Nevertheless, the number of copper alloy artefacts found on terraces T3 and T4, in an area of ca 210 m^2^, is high at about 250 weighing a total of ca 663 g. Most of the artefacts are remnants of the metalworking processes with ca 200 casting drops and small bronze fragments, and 23 other pieces from casting or recycling. In a recent study of the chemistry and isotopic makeup of the Prigglitz-Gasteil metal finds and copper ores, we investigated the *chaîne opératoire* of local copper production and bronze working, as well as the regional distribution networks of metal artefacts. In that study, we concluded that Prigglitz-Gasteil was an active copper mining, metal-making, and importation and recycling site, especially in the late tenth/ninth centuries BC. Prigglitz-Gasteil sourced metal or raw materials were exchanged at least in the Schwarza Valley’s micro-region. However, additional investigations of more distant LBA sites will be necessary to fully explore the extent of exchange and the Prigglitz-Gasteil mining site’s role (Mödlinger et al. 2021).

**Fig. 1:**
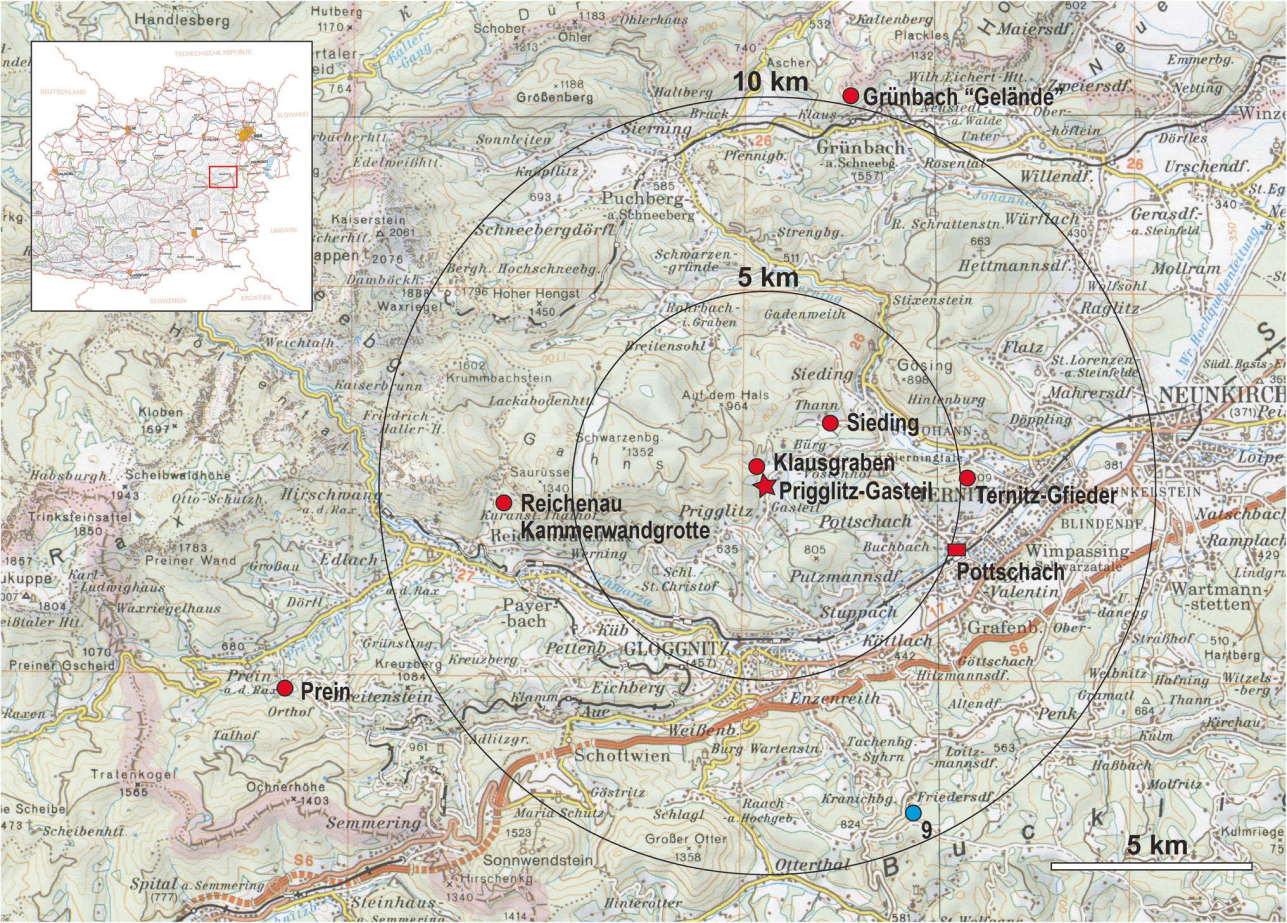
Location of Prigglitz-Gasteil and the surrounding find spots of the analysed finds in this paper (Cartography: © BEV (Bundesamt für Eich- und Vermessungswesen), 2021)

The aim of this paper is to investigate the post-casting treatment of select bronze objects to gain insight into post-casting manufacturing processes and the skills of regional craftsmen (Fig. 1). For this work, a series of 20 copper and bronze objects from the Prigglitz-Gasteil copper mining site, and the surrounding Late Bronze Age dated cemetery, cave, hilltop, and hoards, located within a radius of ca 15 km, were selected for metallographic investigation. The 20 metallographic analyses presented in this paper are compared to objects from the Mahrerdorf Late Bronze Age hoard (Mödlinger and Trebsche 2020).

## Materials and methods

### Selected objects and their site context

**Fig. 2:**
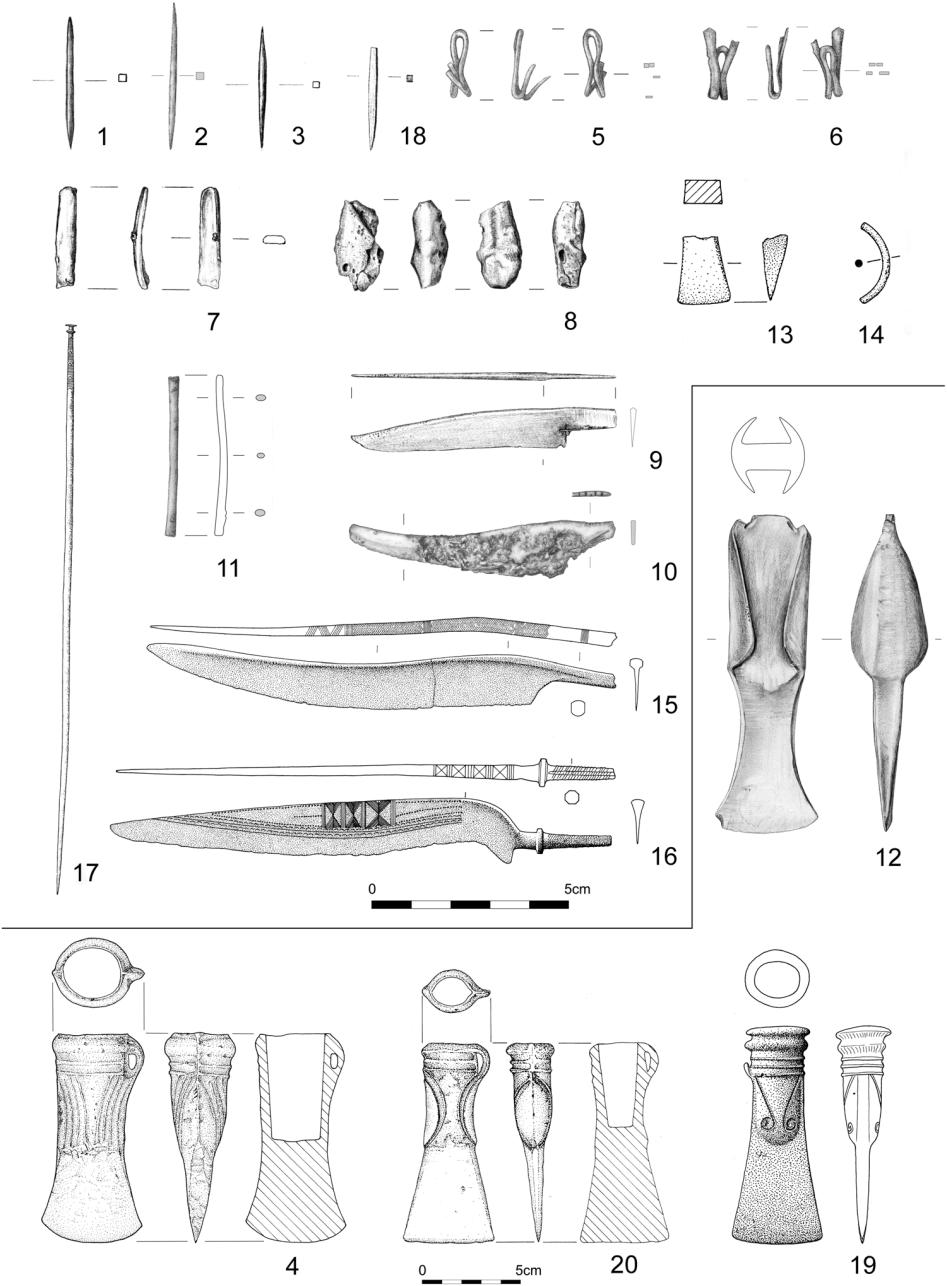
Late Bronze Age objects from southeastern Lower Austria. Numbers 1–3 and 5–11 are from Prigglitz-Gasteil; 4 from Prigglitz-Gasteil, Klausgraben; 12 Grünbach, Gelände; 13–14 Reichenau, Kammerwandgrotte; 15–17 Pottschach; 18 Prein an der Rax; 19 Sieding; and 20 Ternitz, Gfieder The drawings; nos. 1–3, 5–8, 10–12, 18 were done by Daniela Fehlmann and Ulrike Weinberger; 4, 13–14, 20 by Franz Drost; 9, 15–17, 19 are from unknown artist(s). The numbers correspond to those listed in Tables 1, 2, and 3

From the numerous copper alloy fragments found at, and in one instance near, Prigglitz-Gasteil, almost all of the preserved tools with cutting edges or points were selected for metallographic analysis. These tools include a tanged Stillfried-type knife (Fig. 2: 10; cf. Říhovský, 1972, 55–58; Jiráň 2002, 59–60; Veliačik 2012, 305–306), a tanged knife that had been reworked from a fragment (Fig. 2: 9; Říhovský, 1972, 76), and three double-pointed awls (Fig. 2: 1–3). One socketed axe with curved decoration (Fig. 2: 4; cf. Mayer, 1977, 192–198) was found ca 500 m away at Klausgraben. The jewellery selected for analysis included two belt clips (Fig. 2: 5–6), one fragment of a bracelet with a flat cross-section (Fig. 2: 7), and a rod or wand of unknown function (Fig. 2: 11). All the objects are copper-tin alloys except for a casting cake of unalloyed copper (Fig. 2: 8). Strictly speaking, the local production of the bronze artefacts cannot be proven, as chemical and lead isotope analyses have shown that mixing of different copper alloys and recycling played a significant role at the Prigglitz-Gasteil site (Mödlinger et al. 2021).

For comparison to these alloys, objects from several nearby sites were selected. The first is a gravesite at Pottschach located 5 km away from Prigglitz-Gasteil (Kerchler, 1960). It was chosen because the same types and decoration of dress pins are found there (Trebsche and Pucher, 2013, 122, Fig. 7/1–2). From the grave goods, two decorated tanged knives, a Velem-St. Vid type (Fig. 2: 15; cf. Říhovský, 1972, 51–53) and a Baumgarten type (Fig. 2: 16; cf. Říhovský, 1972, 67–71), and one pin with a small vase head (Fig. 2: 17; cf. Říhovský, 1979, 198–207) were studied. The second site, the Kammerwandgrotte cave, located 7 km from the Prigglitz mine at Reichenau an der Rax where there is evidence for Early and LBA activities, including metallurgy (Hottwagner and Lang, 1999), was selected. One chisel fragment (Fig. 2: 13) and one wire fragment (Fig. 2: 14) from the cave were analysed due to their compositional similarity to the copper produced at Prigglitz-Gasteil. These artefacts cannot be precisely dated by find contexts. In the third site, from the LBA mining region of Prein an der Rax, ca 13 km from Prigglitz, a double-pointed bronze tool (Fig. 2: 18) was chosen for study. Smelting activities in this region have been dated by radiocarbon to the Late Urnfield period (ninth century BC; Trebsche, 2015b, 43–47). Fourth, from the metalworking centre likely located at the Gelände near Grünbach hillfort in Schneeberg, one end-winged Haidach type axe (Mayer, 1977, 152–158; Fig. 2: 12) was selected. It belongs to a hoard dating to phase Ha A (ca 1200 to 1080 BC; Trebsche et al., 2019). Fifth, almost all cutting tools (a pickaxe, a chisel, an adze, two winged axes, and three socketed axes) from the Mahrersdorf hoard (Lauermann and Rammer, 2013, pl. 44–47) were sampled. Archaeometallurgical analyses of this hoard have already been published in a separate article (Mödlinger and Trebsche, 2020). And finally, two LBA socketed axes found without context from Sieding (Fig. 2: 19) and at the mountain Gfieder in Ternitz (Fig. 2: 20), respectively, were chosen for comparison to the samples from Prigglitz. The specimen from Ternitz belongs to the type ‘*mit bogenumrandetem Lappendekor und abgesetzter Klinge*’ (Mayer, 1977, 198–199), whereas the other has unique decoration and was classified as a special type (Mayer 1977, 204 no. 1175).

In sum, the objects studied in this paper include four axes, four knives, four awls, one casting cake, two belt clips, one chisel,  two pieces of jewellery (bracelet, pin),  one wire fragment, and a bronze rod/wand (Table 1). X-ray fluorescence and Pb-isotope analyses of ca 125 finds from Prigglitz and the surrounding area, including the objects presented in this paper, are published elsewhere (Mödlinger et al. 2021).

**Table 1:** Metal objects sampled for metallographic analysis from Prigglitz and the surrounding area. Objects in ‘[]’ do not have inventory numbers and are instead labelled with the sampling number. ‘NHM’ corresponds to the Natural History Museum Vienna; ‘LNÖ’, State Collections of Lower Austria; and ‘SL’, Reinhard Lang’s private collection, Gloggnitz. The absolute chronology follows Sperber (2017)

No	Find spot	Context	Object	Museum	Inv. no	Sampling	Relative chronology	Absolute chronology	Type	Reference
1	Prigglitz-Gasteil	Mining site	Double-pointed ‘awl’	LNÖ	UF-22692.1272	Tip	Ha B2-3	960–800 BC	-	Unpublished
2	Prigglitz-Gasteil	Mining site	Double-pointed ‘awl’	LNÖ	UF-22692.1140A	Tip	Ha B2-3	960–800 BC	-	Trebsche / Pucher, 2013, Fig. 19/10
3	Prigglitz-Gasteil	Mining site	Double-pointed ‘awl’	LNÖ	UF-22692.1672	Tip	Ha B2-3	960–800 BC	-	Unpublished
4	Prigglitz-Gasteil, Klausgraben	Isolated find near mining site	Axe (socketed)	SL	[S001]	Edge	Ha B	1080–800 BC	With curved decoration	Trebsche, 2015b, 47 Fig. 2/3
5	Prigglitz-Gasteil	Mining site	Belt clip	LNÖ	UF-22692.1652	End	Ha B2-3	960–800 BC	-	Unpublished
6	Prigglitz-Gasteil	Mining site	Belt clip	LNÖ	UF-22692.1673	End	Ha B2-3	960–800 BC	-	Unpublished
7	Prigglitz-Gasteil	Mining site	Bracelet	LNÖ	UF-22692.1780	End	Ha B2-3	960–800 BC	-	Unpublished
8	Prigglitz-Gasteil	Mining site	Casting cake	LNÖ	UF-22692.675	Border	Ha B2-3	960–800 BC	-	Unpublished
9	Prigglitz-Gasteil	Mining site	Knife	LNÖ	UF-10,964	Edge	Ha B2-3	960–800 BC	(Reworked from a fragment)	Říhovský, 1972, 76 no. 303 pl. 29/303
10	Prigglitz-Gasteil	Mining site	Knife	LNÖ	UF-22692.2188	Edge	Ha B2-3	960–800 BC	Type Stillfried	Trebsche, 2015b, Fig. 2/6
11	Prigglitz-Gasteil	Mining site	Rod/wand	LNÖ	UF-22692.912	End	Ha B2-3	960–800 BC	-	Trebsche / Pucher, 2013, Fig. 19/6
12	Grünbach, Gelände	Hilltop settlement	Axe (end-winged)	LNÖ	UF-19,452	Edge	Ha A	1200–1080 BC	Type Haidach	Trebsche et al., 2019, 561 Fig. 5
13	Reichenau, Kammerwandgrotte	Cave	Chisel	LNÖ	[S041]	Edge	LBA?	1330–800 BC?	-	Hottwagner / Lang, 1999, 779 Fig. 749
14	Reichenau, Kammerwandgrotte	Cave	Wire (bent)	LNÖ	[S013]	End	LBA?	1330–800 BC?	-	Hottwagner and Lang, 1999, 779 Fig. 748
15	Pottschach	Cemetery	Knife	NHM	72.485	Edge	LBA	1330–800 BC	‘Griffdornmesser vom Typ Velem St. Vid’	Říhovský, 1972, 52 no. 179 pl. 17/179
16	Pottschach	Cemetery	Knife	NHM	72.484	Edge	Ha B2-3	960–800 BC	‘Griffdornmesser vom Typ Baumgarten’	Říhovský, 1972, 68 no. 274 pl. 26/274
17	Pottschach	Cemetery	Pin	NHM	72.488	Shaft	Ha B2-3	960–800 BC	‘Nadel mit kleinem Vasenkopf’	Říhovský, 1979, 203 no. 1706 pl. 62/1706
18	Prein an der Rax	Smelting site	Double-pointed ‘awl’	LNÖ	UF-9958	Tip	Ha B2-3	960–800 BC	-	Hampl and Mayrhofer, 1963, 52 (Prein V)
19	Sieding	Isolated find	Axe (socketed)	LNÖ	UF-5098	Edge	Ha B	1080–800 BC	(Special type)	Mayer, 1977, 204 no. 1175 pl. 84/1175
20	Ternitz, Gfieder	Isolated find from hilltop settlement	Axe (socketed)	SL	[S042]	Edge	Ha B2-3	960–800 BC	‘mit bogenumrandetem Lappendekor und abgesetzter Klinge’	Lang, 2000, 599 Fig. 474

### Methodology

Microstructural characterization of the objects and chemical analyses using energy-dispersive X-ray spectroscopy (EDXS) were performed on freshly polished cross-sections taken at the edges of the blades for axes, chisels, and knives; tip for awls; centre of wires and bracelets; and end of belt clips and pins (Fig. 2). Further, X-ray fluorescence (XRF) chemical and high-resolution multi-collector inductively coupled mass spectrometry (HR-MC-ICP-MS) Pb isotope analyses were later carried out on the same and on freshly polished samples (see Mödlinger et al. 2021). The EDXS analyses were performed using a JEOL JSM-6460LV SEM with an Oxford Instruments SDD XMax 20 under high vacuum at IRAMAT-CRP2A, Bordeaux, France. The SEM was calibrated using the internally provided software database standards, as well as certified pure Si and Co standards for quantification. The results shown are the mathematical average of 5–8 spectra of approximately 200 × 600 μm taken for 60 s each. The presence of minor and trace elements was supported by their detection in higher amount in the corrosion layers. The SEM-EDXS analyses were used to identify different intermetallic compounds, inclusions, and the absence or presence (qualitative) of sulphur (S), which was not detected by XRF. The qualitative presence of each alloying element was classified as major with wt.% > 1, minor between 1 and 0.3, and trace at < 0.3; their presence was normalized and is given in wt.% in Table 2.

Chemical analysis was carried out on drilling samples using an ARL Quant’X (Thermo Fisher Scientific) XRF (bulk analyses) at 28 kV (with Pd filter) and 50 kV (with Cu filter), and on the surfaces of the samples polished for metallographic study using a Fischerscope X-ray XAN 150 (W-band) (point measurements) at 50 kV (Al-filter) using a 1 mm collimator SD-detector for 50 s. The measuring time/spot of 1–2 measurements/sample depended on the sample size for both instruments. Each analysis was performed at the CEZA-laboratory in Mannheim, Germany, for the bulk and points, respectively. Quantification of the resultant analyses closely followed the procedure described in Lutz and Pernicka (1996). Manganese, Co, Zn, Se, Cd, Te, and Bi were below the detection limit of the Fischerscope, and, since S was only measured with EDXS, the results shown in Table 2 should be considered qualitative. There are notable differences between the ARL Quant’X and Fischerscope results (e.g. sample nos. 19a and b), which are due to all-inclusive bulk versus point measurements, the nature of the sample (drilling vs. metallographic cross-section), and the presence of inhomogeneities and corrosion. The error rate for both techniques is 5–10% for the major elements, and even lower for Cu, and 20–50% for minor and trace elements.

**Table 2:** The elemental percentages of each sample are given in wt.%. Manganese, Co, Zn, Se, Cd, Te, and Bi were not detected in the samples analysed with the Fischerscope (FS). All other samples show ≤ 0.005 Mn, Se, and Te, 0.01 Co, < 0.1 Zn, < 0.01 Cd, and < 0.06 Bi. Use of the Fischerscope is indicated by FS. An asterisque (*) indicates drilling samples analysed by ARL Quant’X XRF. Noteworthy are the differing amounts of Sn in the socketed axe from Sieding that appear with different analytical methods (see ‘Sieding, inv. no. UF-5098, socketed axe’)

No	Site	Object	Inv.no	Cu	Fe	Ni	As	Ag	Sn	Sb	Pb	FS
1	Prigglitz-Gasteil	Double-pointed ‘awl’	UF-22692.1272	89	n.d	n.d	n.d	n.d	**11.0**	n.d	n.d	x
2	Prigglitz-Gasteil	Double-pointed ‘awl’	UF-22692.1140A	90	n.d	n.d	n.d	n.d	**9.5**	0.12	n.d	x
3	Prigglitz-Gasteil	Double-pointed ‘awl’	UF-22692.1672	86	n.d	0.07	n.d	n.d	**13.7**	0.10	0.08	x
4	Prigglitz-Gasteil, Klausgraben	Axe (socketed)	[S001]*	91	< 0.05	0.12	0.021	0.008	**8.5**	0.092	0.011	
5	Prigglitz-Gasteil	Belt clip	UF-22692.1652	91	0.22	0.07	0.03	n.d	**8.3**	0.24	n.d	x
6	Prigglitz-Gasteil	Belt clip	UF-22692.1673	88	0.06	0.28	0.32	0.28	**10.3**	0.37	0.34	x
7	Prigglitz-Gasteil	Bracelet	UF-22692.1780	87	0.13	0.04	n.d	n.d	**12.8**	0.13	n.d	x
8	Prigglitz-Gasteil	Casting cake	UF-22692.675*	100	0.11	0.06	0.012	0.010	**0.017**	0.095	0.012	
9	Prigglitz-Gasteil	Knife	UF-10,964	84	0.73	0.05	n.d	n.d	**15.3**	n.d	0.14	x
10	Prigglitz-Gasteil	Knife	UF-22692.2188	89	0.19	0.14	0.14	n.d	**9.9**	0.41	n.d	x
11	Prigglitz-Gasteil	Rod / wand	UF-22692.912	87	n.d	0.03	n.d	n.d	**11.9**	0.06	0.61	x
12	Grünbach, Gelände	Axe (end-winged)	UF-19,452	89	0.09	0.40	0.51	0.15	**9.1**	0.52	0.19	x
13	Reichenau, Kammerwandgrotte	Chisel	[S041]	91	n.d	0.15	0.19	0.08	**7.5**	0.25	0.39	x
14	Reichenau, Kammerwandgrotte	Wire (bent)	[S013]	88	0.14	0.06	0.06	n.d	**12.0**	0.20	0.05	x
15	Pottschach	Knife	72,485*	89	0.36	0.06	0.132	0.087	**9.2**	1.24	0.019	
16	Pottschach	Knife	72,484*	89	0.05	0.07	0.021	0.016	**10.6**	0.214	0.020	
17	Pottschach	Pin	72,488*	90	0.10	0.07	0.015	0.020	**9.2**	0.130	0.007	
18	Prein an der Rax	Double-pointed ‘awl’	UF-9958	94	0.08	0.05	n.d	n.d	**5.2**	0.10	0.69	x
19a	Sieding	Axe (socketed)	UF-5089 [S022]*	92	0.11	0.13	0.051	0.011	**7.7**	0.095	0.102	
19b	Sieding	Axe (socketed)	UF-5089 [S039]	89	0.11	0.11	0.06	n.d	**10.3**	0.14	0.09	x
20	Ternitz, Gfieder	Axe (socketed)	[S003]*	91	0.16	0.06	0.01	0.005	**8.7**	0.02	0.005	

Characterization of the sample’s microstructure was performed on prepared cross-sections. Each sample was mounted in cold acrylic resin and polished using 400–1200 SiC papers, followed by a diamond suspension paste of up to 0.25 μm granulometry. The samples were characterized using optical microscopy in both bright and dark fields, chemically analysed using EDXS, and then etched for metallographic examination using aqueous ferric chloride and Klemm II to show greater detail. While aqueous ferric chloride produces a grain contrast, Klemm II is a colour etchant, which colours grains depending on their orientation; segregation also becomes visible and intermetallics are not etched. The total amount of deformation applied to each sample was calculated by measuring the shape factor (SF) of the CuS or CuFeS inclusions (see Mödlinger and Piccardo 2013). Vickers hardness measurements were carried out using a Leitz Durimet 72-1b instrument at 100 g load over 10 s. An FT-9929195 test block from Future-Tech-Corporation was used as a standard.

## Results

In the following, the results are focused on the metallographic analyses. The chemical analyses are thoroughly discussed elsewhere (Mödlinger et al. 2021). However, the chemical compositions of the finds discussed in this paper are provided in Table 2.

### Axes and chisel

#### Prigglitz-Gasteil, ID S001, socketed axe

**Fig. 3:**
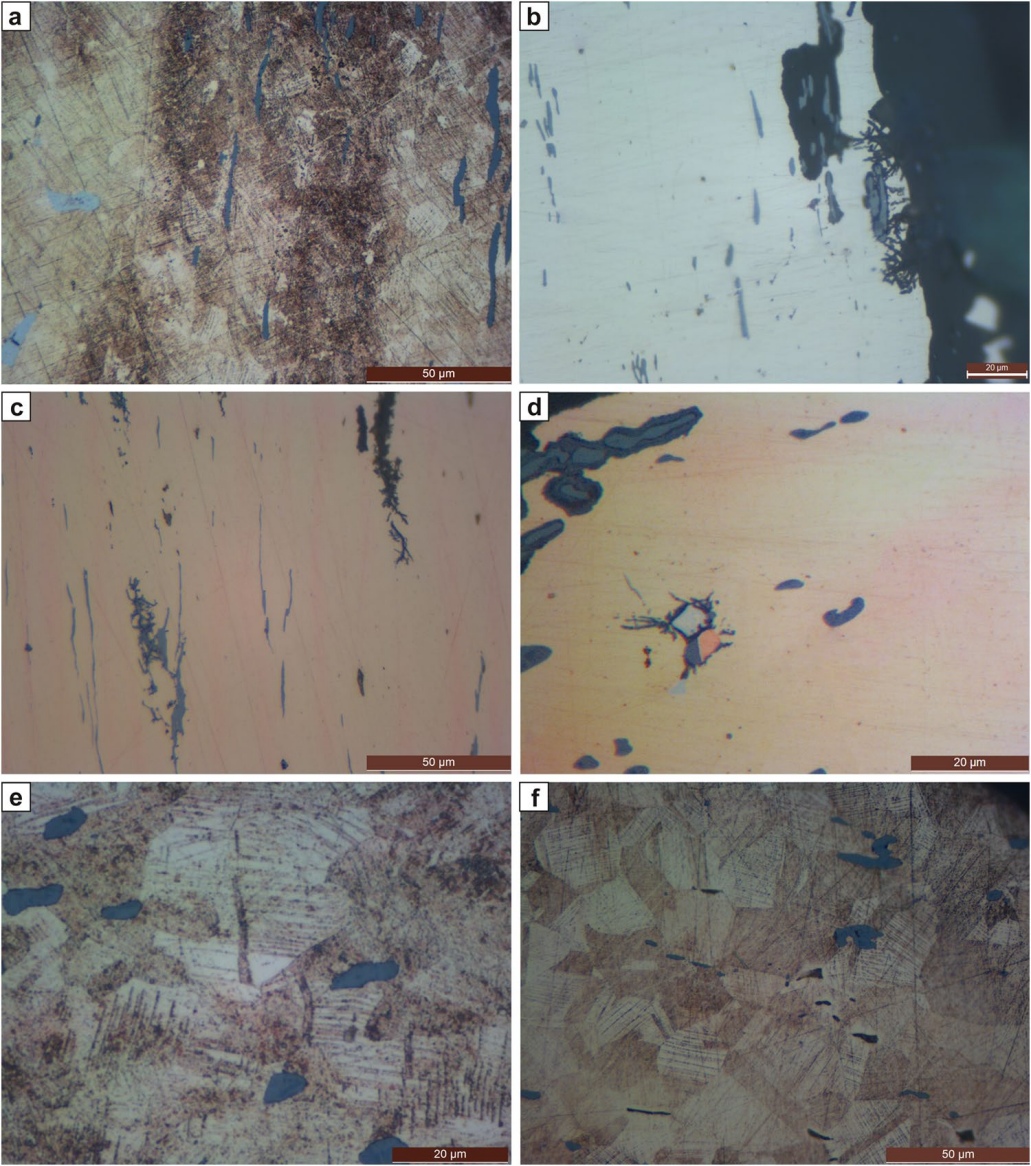
Microstructures. Axe ID S001 from Prigglitz-Gasteil: **a** SEM image showing coring, CuS-inclusions (dark grey), and broken (α + δ) eutectoid (light grey). Winged axe inv.no. UF-19.452 from Grünbach: **b** Unetched, in polarized light. Bacterial induced corrosion is visible. Chisel ID S041 from Kammerwandgrotte: **c** Unetched. Coring is visible as massively elongated CuS-inclusions. The corrosion follows the microstructural features inter- and intracrystallinearly. Socketed axe ID S042 from Ternitz: **d** Unetched. Coring is visible. At the centre, one can see (α + δ) eutectoid (light grey) with surrounding inter- and intracrystalline corrosion, under which are cuprite (dark grey, below the eutectoid) and a copper inclusion. The CuFeS-inclusions (dark grey) are not significantly deformed. **e** Etched with ferric chloride. Note the deformed grains of α-solid solution with deformed twins and strain lines. Socketed axe inv.no. UF-5098 from Sieding: **f** Etched with ferric chloride. Note the deformed grains of α-solid solution with deformed twins and strain lines

A sample was taken on the edge of the axe’s blade. Analysis of the sample showed 8.5 wt.% Sn and 0.6% S. Nickel was also present at about 0.1% with Fe, As, Sb, Ag, and Pb in trace amounts. The unetched sample shows CuS-inclusions with about 60–70% deformation. Etching with ferric chloride reveals coring (zones with 9 to 15% Sn are clearly distinguishable) and small, equiaxed polyhedric grains. The grains are deformed and show strain lines and annealing twins. In the matrix, (α + δ) eutectoid is present; due to its brittleness and the applied deformation, there are many cracks (Fig. 3a). Their presence in the axe indicates that annealing took place at relatively low temperatures (i.e. < 520 °C), which counterintuitively still permitted the formation of new grains. Corrosion in the sample mainly consists of copper oxide inclusions (cuprite) and copper carbonates. There are also corrosion layers of tin oxides on the object’s surface, which are typical for bronze. Hardness measurements give up to 297 HV values in tin-richer zones and 245 HV in zones with less tin. In the more homogenous zone at the very edge, hardness reaches 254 HV, while the sample’s core is between 206 and 245 HV.

#### Grünbach am Schneeberg, inv. no. UF-19.452, median-winged axe

The median-winged axe-type Freudenberg from Grünbach am Schneeberg was sampled on the edge and found to contain 9% Sn, 0.5% of As and Sb each, as well as 0.1% Ag, 0.4% Ni, and 0.2% Pb. Iron is present in traces. The unetched sample shows ca 30–40% deformed, light grey CuS-inclusions. Etching with ferric chloride reveals coring (indicating a non-complete homogenization) as well as slightly deformed polyhedric grains with twins and strain lines. Some eutectoid is present. Corrosion can be found inter- and intracrystalline. On the edge of the sample, corrosion follows the dendritic structure. Also, bacterial-induced corrosion was noted (see Piccardo et al., 2013) (Fig. 3b). Copper oxide (mainly cuprite) and copper carbonate layers cover the surface of the sample. Hardness measurements give about 151–221 HV values, with a harder edge than in the sample’s core.

#### Kammerwandgrotte, ID S041, chisel

The chisel was sampled on its edge and found to contain 7.1–8% Sn (depending on the analytical method used), 0.2% As, 0.1% Ni, 0.2% Sb, 0.4% Pb, and some S. The S was under the detection limit of the EDXS for bulk analyses but was detected in the CuS-inclusions. Iron and Ag are present in traces, and the unetched sample shows ca 90% deformed light grey CuS- and globular Pb-inclusions (Fig. 3c). Etching with ferric chloride revealed slight coring, indicating incomplete homogenization, and fine, slightly deformed, polyhedric grains with strain lines and twins. There is also (α + δ) eutectic, and corrosion is present both inter- and intracrystallinearly. Copper oxide crystals (cuprite; dark red in polarized light) and alternating copper oxides and carbonate layers cover the surface of the sample. Tin oxides are present, as are P, Si, and Ca, which derive from the soil. Hardness measurements give about 202–237 HV values, with a harder edge than in the core of the sample.

#### Ternitz (Gfieder), ID S042, socketed axe

The socketed axe was sampled on its edge and found to contain 10.8% Sn using EDXS and 8.7% with XRF, and 0.7% S and about 0.16% Fe. Sulphur and Fe are mainly present in the CuFeS-inclusions. Nickel, As, Ag, Sb, and Pb are present in trace amounts. The unetched sample shows slightly deformed CuFeS-inclusions, which indicate a total deformation of about 10–20% in the sampled area (Fig. 3d). Etching the sample with ferric chloride revealed an inhomogeneous microstructure — tin-rich areas contain up to 15% Sn, tin poor areas up to 6% — and severely deformed equiaxed grains of α-solid solution (Fig. 3e). The grains show deformed annealing twins as well as strain lines, and (α + δ) eutectoid is present. The deformation and presence of eutectoid indicate annealing at low temperature (< 520 °C) or short annealing at higher temperatures with a final heavy deformation. Some copper drops are also visible in the matrix, and the corrosion follows both the original dendritic structure and, in small areas, the grain boundaries and the intracrystalline structures of the α-solid solution equiaxed grains. Both copper oxides (cuprite) and copper carbonates (mainly azurite and malachite) are visible in polarized light, as is cuprite as inclusions. The hardness values are 206–242 HV on the very edge and slightly lower (193–221 HV) 3 mm inward, corresponding to high final deformation.

#### Sieding, inv. no. UF-5098, socketed axe

The socketed axe was sampled on its edge and contained 7.7–11.4% Sn, 0.5% S, and about 0.1% Fe, Ni, Sb, and Pb. Sulphur and Fe are mainly present in the CuFeS-inclusions, and As and Ag are in trace amounts. The Sn composition varied by sample type, with XRF of the drilling samples showing 7.7% and surface analyses of the metallographic sample at about 10.3%, similar to the 11.4% result from the EDXS. The unetched sample shows slightly deformed CuFeS-inclusions, which indicate a total deformation of about 10–20%. Lead is present in small inclusions. Etching the sample with ferric chloride revealed coring and a very fine grain structure (grain sizes smaller than 10, according to ASTM) (Fig. 3f). The equiaxed grains of α-solid solution showed twins and strain lines. While the grains show slight deformation on the very edge, they are slightly more deformed in the sample’s core. The corrosion follows the inter- and intracrystalline structures of the grains. Calcium, Cl, Si, and tin oxides were present in the corrosion, with the former three deriving from the surrounding burial soil. The most significant part of the corrosion is copper oxides (cuprite) and carbonates of mainly azurite and malachite. The hardness values of 213–216 HV in the sample’s core are higher than on the very edge (166–170 HV). These hardness values also correspond with the more deformed grains in the sample’s core.

### Knives

#### Pottschach, inv. no. 72.484, knife

**Fig. 4:**
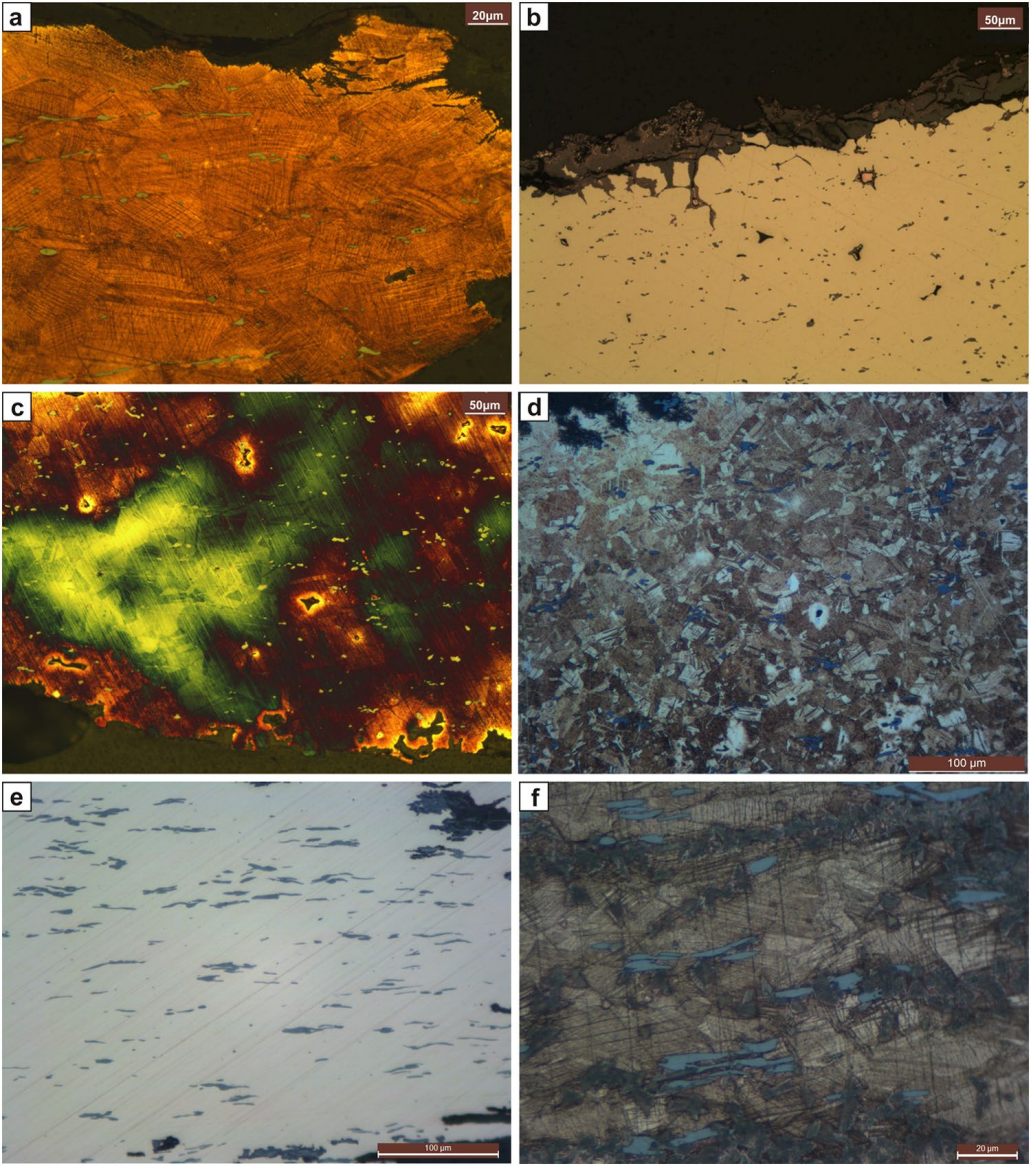
Microstructures. Knife inv.no. 72.484 from Pottschach: **a** Etched with ferric chloride. Note the elongated CuFeS-inclusions and the deformed grains of α-solid solution with deformed twins and strain lines. **b** Knife inv.no. 72.485 from Pottschach: **b** Unetched. Note the copper drops in the matrix, surrounded by corrosion. **c** Etched with ferric chloride. Knife inv.no. 10.964 from Prigglitz: **d** Etched with ferric chloride. Note the slightly deformed twins and strain lines of the polyhedric grains of α-solid solution. Also, the (α + δ) eutectoid is present. Knife inv.no. UF-22692.2188 (22.692) from Prigglitz: **e** Unetched. Slight coring is visible. **f** Etched with ferric chloride. Note the deformed polyhedric grains of α-solid solution with twins and strain lines

The knife was sampled on its edge and found to contain 11% Sn, 0.2% Sb, and 0.4% S. Sulphur and small amounts of Fe are mainly present in CuFeS-inclusions. Iron, Ni, As, Ag, and Pb are present in trace amounts. The sample was taken from the centre of the blade’s edge. The unetched sample shows elongated CuFeS-inclusions, indicating a total deformation of 30–40% at the very edge and about 10–20% towards the core in the sample. Etching the sample with ferric chloride revealed equiaxed grains of α-solid solution with twins (Fig. 4a). Only at the very edge, congruent with the significantly more deformed CuFeS-inclusions in this area did the grains show deformation and show strain lines with slight coring.

As the final working step, the edge of the chisel was cold deformed. The corrosion follows both the deformed α-solid solution equiaxed grains’ grain boundaries and the intragranular annealing twins. Under polarized light, the sample showed both copper oxides (cuprite) and carbonates (mainly azurite and malachite). The hardness values of 206–228 HV on the very edge correspond with microstructural observations (higher levels of total deformation of CuFeS-inclusions and grain deformation and strain lines) and the relatively high amount of Sn. The core of the sample dissimilarly showed 132–151 HV.

#### Pottschach, inv. no. 72.485, knife

The knife was sampled on its edge and found to be a tin-antimony bronze containing mean values of 9% Sn, 1.2% Sb, 0.1% As, 0.7% S, and 0.4% Fe. Sulphur and Fe are mainly present in CuFeS-inclusions, and Ni, Ag, and Pb are present in trace amounts. The sample was taken from the edge at the centre of the blade. The unetched sample shows slightly elongated CuFeS-inclusions, which indicate a total amount of deformation of 20–30% at the very edge in the sampled area, and about 10–20% in the core. Etching the sample with ferric chloride revealed heavy coring and large equiaxed grains (ca 30–60 μm in diameter) of α-solid solution with twins (Fig. 4c). As the final working step, the knife’s edge was annealed. Some copper drops are visible in the matrix (Fig. 4b). The corrosion follows the grain boundaries of the α-solid solution equiaxed grains. According to their colour under polarized light, copper carbonates (mainly azurite and malachite) are the main corrosion products. The hardness values of 128–143 HV confirm the microstructural observations; though the edge received a higher amount of total deformation, the annealing following the cold deformation resulted in an equally low hardness throughout the sample.

#### Prigglitz, inv. no. 10.964, knife

X-ray fluorescence analyses could not be carried out with accuracy due to the presence of corrosion; however, it is worth pointing out that traces of Ni and Pb were detected. The following compositional percentages derive from the SEM-EDXS analyses carried out on the polished metallographic sample’s surface. The knife was formed from a tin-bronze containing mean values of 13.5% Sn and 0.6% S that were mainly present in CuFeS-inclusions. The sample was taken from the centre of the blade. The unetched sample shows elongated CuFeS-inclusions, which indicate a total amount of deformation of 60–70% at the very edge and about 10–20% towards the core. Etching the sample with ferric chloride revealed coring and very fine polyhedric grains of α-solid solution with twins and strain lines (Fig. 4d). The grains are severely deformed along the edge, and (α + δ) eutectoid is present, indicating low temperature (< 520 °C) or shorter annealing at higher temperatures took place. The applied deformation did not result in a broken eutectoid. The SEM images showed tiny, globular Pb-inclusions throughout the matrix. Corrosion in the sample follows intracrystalline structures to a smaller degree, the dendritic features. Elements such as Al, Si, and P were present and derived from the soil. Apart from the copper oxides and carbonates, tin oxides are also present. The hardness values of 245–254 HV on the very edge and 181–199 in the sample’s core confirm the microstructural observations.

#### Prigglitz, inv. no. UF-22692.2188 (22.692), knife

X-ray fluorescence analyses of this sample should be considered qualitative, as some corrosion was present. The knife was formed from tin-bronze containing mean values of 10% Sn with about 0.4% Sb, 0.2% Fe, and 0.1% Ni and As. The S and Fe are present in CuFeS-inclusions. The sample was taken from the blade. The unetched sample shows slightly elongated CuFeS-inclusions, which indicate a total amount of deformation of 30–40%. Etching the sample with ferric chloride revealed coring and significantly deformed polyhedric grains of α-solid solution with twins and strain lines (Fig. 4e–f). As the final working step, the edge of the knife was cold deformed. The corrosion follows both the previous as-cast structure (dendrites) as well as the grain boundaries of the α-solid solution equiaxed grains. According to their colour under polarized light, copper carbonates (mainly azurite and malachite) are the main corrosion products. The hardness values of 193–274 HV, with 274 HV on the very edge, make this the highest hardness value of the four knives.

### Awls

#### Prigglitz, inv. no. UF-22692.1140A, awl

**Fig. 5:**
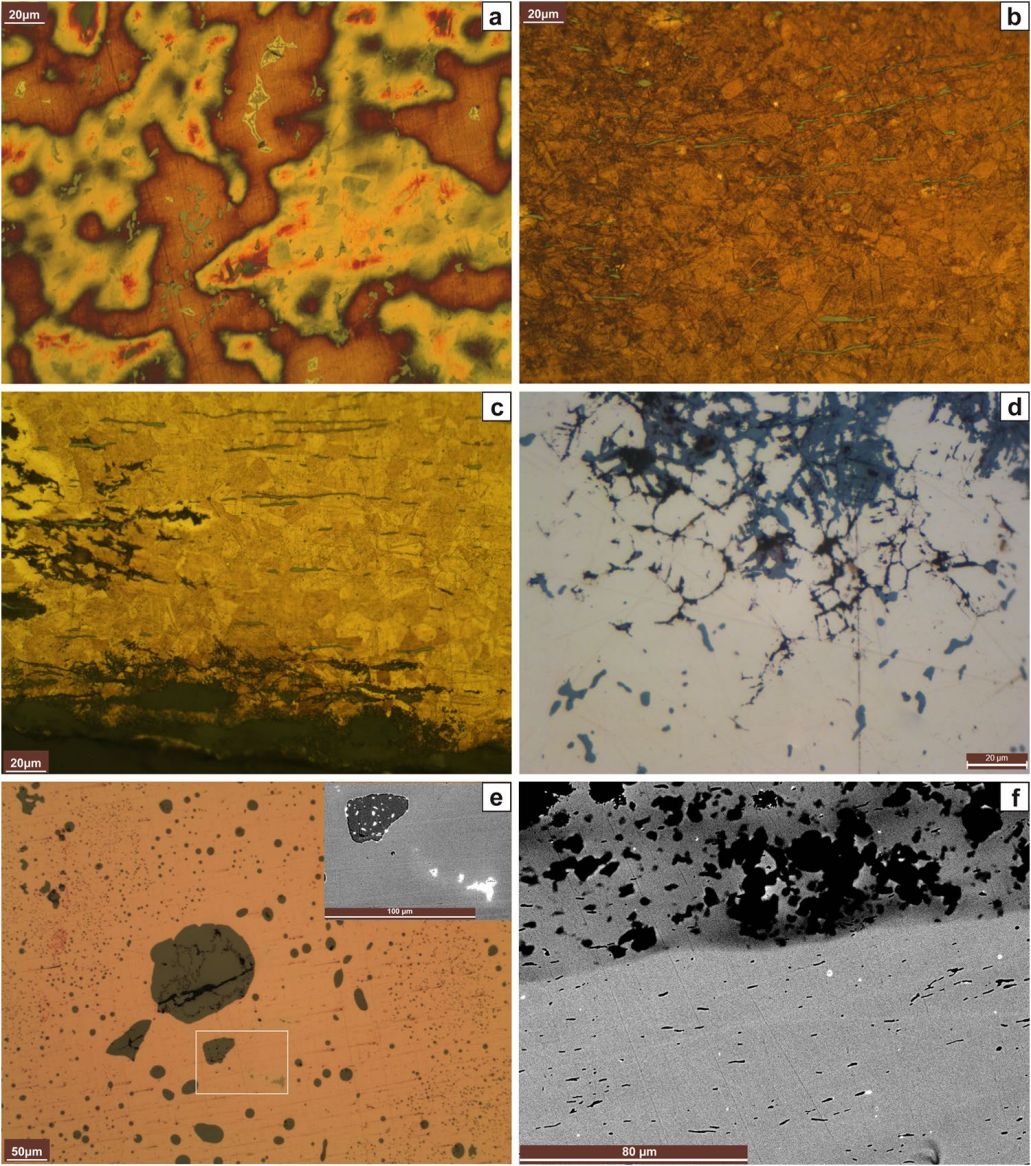
Microstructures. Awl inv.no. UF-22692.1140A from Prigglitz: **a** Etched with Klemm II. Note coring and (α + δ) eutectoid. Awl inv.no. 1272 from Prigglitz: **b** Etched with ferric chloride. The CuS-inclusions are elongated. Awl inv.no. UF-22692.1672from Prigglitz: **c** Etched with ferric chloride. Note the elongated CuS-inclusions. Awl inv.no. UF-9958 from Prein: **d** Unetched. Note the corrosion, outlining the microstructural features. Casting cake inv. no. UF-22692.675 from Prigglitz. **e** Unetched. Note the many CuS-inclusions and, in the centre, some CuSb-inclusions in the SEM image. White inclusions are rich in Sb (ca 35%) as well as Ag and As (below 1%). Belt clip inv.no. UF-22692.1673 from Prigglitz: **f** Etched with Klemm II. Heavy coring is visible

The awl is made of tin-bronze containing mean values of 9.5% Sn, 0.1% Sb, and 0.7% S, which is mainly present in CuS-inclusions. The tip of the awl was sampled longitudinally and transversally (cross-section). The unetched longitudinal sample showed slightly elongated CuS-inclusions, indicating a total amount deformation of 20–30%, while the cross-section only shows light deformation at a maximum of 15%. The (α + δ) eutectoid of the Cu-Sn system is visible in the unetched sample, and once etched with ferric chloride coring and fine, deformed equiaxed grains of α-solid solution with twins and strain lines were visible (Fig. 5a). The presence of (α + δ) eutectoid indicates short, low-temperature annealing, followed by cold deformation. As the final working step, the tip of the awl was cold deformed. Corrosion in the sample follows the grain boundaries of the α-solid solution equiaxed grains, and it expands intracrystallinearly. According to their colour under polarized light and the EDXS analyses, copper carbonates (mainly azurite and malachite) are the main corrosion products on the awl’s surface, while copper (mainly cuprite) and tin oxides are visible in the inter- and intracrystallinearly. The hardness values of 193–228 HV are relatively high for a 9.5% tin-bronze and correspond with the deformation applied in the final working step.

#### Prigglitz, inv. no. UF-22692.1272, awl

The awl was formed from tin-bronze containing mean values of 11% Sn and 0.3% S, which was mainly present in CuS-inclusions. The tip of the awl was sampled longitudinally, and unetched showed slightly elongated CuS-inclusions, indicating a total amount of deformation of 40–50%. After etching with Klemm II heavy coring was revealed, indicating an inhomogeneous alloy (Fig. 5b). Both Klemm II and ferric chloride etched surfaces showed small, slightly deformed equiaxed grains of α-solid solution with twins as well as strain lines. No (α + δ) eutectoid of the Cu-Sn system was visible, indicating that the awl underwent longer, or more frequent annealing followed by cold deformation. In the final working step, the tip of the awl was cold worked. The corrosion follows the dendritic structure. According to the corrosion colours under polarized light, copper carbonates (mainly azurite and malachite) are the main products on the awl’s surface. No inter- or intracrystalline corrosion was noted. The hardness values of 187–206 HV are relatively high for a 10% tin-bronze and correspond with the deformation applied in the final working step.

#### Prigglitz, inv. no. UF-22692.1672, awl

The awl was formed from tin-bronze containing mean values of 14% Sn, 0.1% Sb, and 0.5% S, which was mainly present in CuS-inclusions with low amounts of Ni. Nickel and Pb are present in trace amounts. The tip of the awl was sampled longitudinally, and unetched showed severely elongated CuS-inclusions, indicating a total amount of deformation of 70–80% (Fig. 5c). After etching the sample with Klemm II and ferric chloride, light coring was revealed, indicating an almost homogenous alloy. Both Klemm II and ferric chloride developed small, slightly deformed equiaxed grains of α-solid solution with twins and strain lines. No (α + δ) eutectoid of the Cu-Sn system remained, indicating more prolonged or more frequent annealing followed by cold deformation. In the final working step, the tip of the awl was cold worked. Half of the sample is massively corroded. The corrosion follows the grain boundaries and, intra-granularly, along the dislocations of single grains (both twins and strain lines). According to the corrosion colours under polarized light, copper carbonates (mainly azurite and malachite) are the main corrosion products on the awl’s surface. Within the corrosion, there was also SnO that was measured by EDXS. Copper oxides (cuprite) can be found in the centre of the sample. The hardness values of 245–274 HV for the sample are the highest measured in this study. They are related to both the high amount of Sn in the alloy and the intense cold deformation applied in the final step of production.

#### Prein, inv. no. UF-9958, awl

The awl is tin-bronze and contains mean values of 5% Sn and 0.7% Pb. Sulphur is mainly present in the CuFeS-inclusions, and Fe in trace amounts. The sample was cut transversally from a fragment of the awl. Unetched, the sample shows slightly elongated CuFeS-inclusions, indicating a total amount of deformation of 0–20%, which is not surprising for the cut. The total amount of longitudinal deformation could not be measured. Etching the sample with ferric chloride revealed coring and small, deformed equiaxed grains of α-solid solution with twins and strain lines. The corrosion follows the grain boundaries of the α-solid solution equiaxed grains and also expands intracrystallinearly (Fig. 5d). According to the sample’s colour under polarized light, copper carbonates (mainly azurite and malachite) are the main corrosion products on the awl’s surface, while copper (mainly cuprite) and tin oxides are visible inter- and intracrystallinearly. The low hardness values of 160–181 HV correspond with the alloy composition.

### Other objects

#### Prigglitz, inv. no. UF-22692.675, casting cake

The casting cake consists of rather pure copper with only 1.7% S, 0.1% Fe, and small amounts of Sb (0.1%). Other elements, such as Ni, Ag, As, and Pb, are present in trace amounts. The highly porous as-cast shows a homogenous copper matrix without any coring or dendrites. No cuprite was noted under polarized light; however, CuO — mainly carbonates — is present in the corrosion and on the casting cake’s surface. Globular, black CuS-inclusions — some of them containing up to 2% O and/or up to 1% Sb — are distributed in various sizes all over the sample’s surface. There are small, white inclusions that mainly contain Sb (35%) and Ag and As below 1% (Fig. 5e). Hardness values are around 96–105 HV.

#### Prigglitz, inv. no. UF-22692.1673, belt clip

The belt clip is made of tin-bronze with about 7.5% Sn, 0.7% Sb, and 0.2% S. The XRF sample contained corrosion, so preference should be given to the SEM-EDXS chemical data; however, it is important to note that with XRF, 0.3% Ni, As, Ag, and Pb were detected. One end of the belt clip was pinched off, and the cross-section of the belt clip englobed in acrylic resin. The unetched sample revealed lightly deformed CuS-inclusions of about 20–30% of total deformation. Some of the inclusions also contain up to 2% of Sn. Etching the sample with Klemm II revealed heavy coring, indicating a non-homogenous alloy (Fig. 5f). The etchant developed undeformed, equiaxed grains of α-solid solution with twins of about ca 30 × 40 μm in diameter. No (α + δ) eutectoid of the Cu-Sn system remained, indicating longer or more frequent annealing followed by cold deformation. In the final working step, the belt clip was shortly annealed. In the corrosion, Ni and Sb were enriched. The corrosion follows both the dendritic structure and grain boundaries and intracrystalline dislocations (annealing twins and strain lines) of single grains. The hardness values of 92–128 HV correspond to annealing applied as a final working step.

#### Prigglitz, inv. no. UF-22692.1652-A, belt clip

**Fig. 6:**
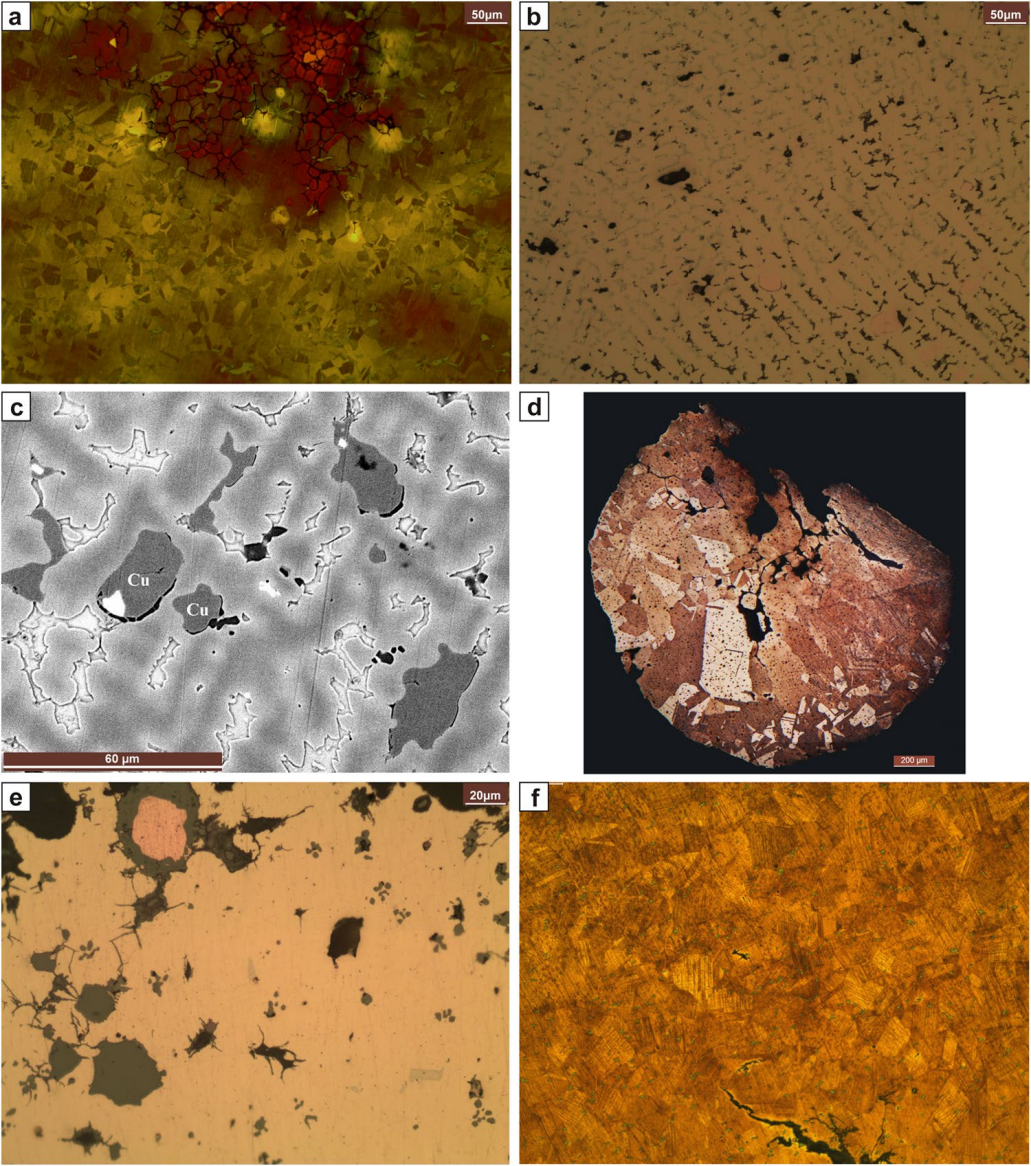
Microstructures. Belt clip inv.no. UF-22692.1652 from Prigglitz: **a** Etched with Klemm II. Coring and fine equiaxed grains of α-solid solution with annealing twins. Rod inv.no. UF-22692.912 from Prigglitz: **b** Etched with Klemm II; much (α + δ) eutectoid and Cu-drops are visible. **c** SEM image. Note the Cu-drops and the massive (α + δ) eutectoid. Wire ID S013 from Kammerwandgrotte: **d** Etched with ferric chloride. Complete cross-section. Note the smaller grain size on the outside. Prigglitz, bracelet (inv.no. UF-22692.1780): **e** Unetched. Note the copper drop and the presence of (α + δ) eutectoid. The CuFeS-inclusions are not deformed. Pottschach, pin (inv.no. 72.488): **f** Etched with ferric chloride

The belt clip is made of tin-bronze with about 8% Sn, 0.6% S (EDXS), 0.2% Fe, and 0.4% Sb. Nickel and As are also present in trace amounts, and Ag and Pb were not detected. One end of the belt clip was pinched off, and the cross-section of the belt clip englobed in acrylic resin. Unetched, the sample revealed elongated CuFeS-inclusions of about 30–40% of total deformation. The inclusions also contain lesser amounts of Sb. Etching the sample with Klemm II revealed heavy coring, indicating an inhomogeneous alloy. The etchant developed small, ca 25 × 35 μm in diameter, equiaxed grains of α-solid solution with twins (Fig. 6a). No (α + δ) eutectoid of the Cu-Sn system remained, indicating longer or more frequent annealing followed by cold deformation. As the final working step, the belt clip was shortly annealed. Under polarized light and supported by EDXS analyses, copper carbonates and oxides are present, as are various tin oxides. Corrosion in the sample follows both the dendritic structure and grain boundaries and intracrystallinearly between the dislocations (annealing twins) of single grains. The hardness values of 96–110 HV correspond to annealing in the final working step.

#### Prigglitz, inv. no. UF-22692.912, rod/wand

The rod is made of tin-bronze with about 12% Sn, 0.5% S (EDXS), and 0.6% Pb. Nickel and Sb are present in trace amounts, while Fe, As, and Ag were not detected. A cross-section of one end of the rod was englobed in acrylic resin. The unetched sample shows a porous, as-cast, dendritic microstructure with a few globular CuS-inclusions and high amounts of (α + δ) eutectoid in the Sn-rich zones of the alloy. The eutectoid may also contain some Fe. Of particular importance are the drops of pure Cu surrounded by CuS-inclusions (Fig. 6b–c). These Cu-drops indicate that not all of the Cu was dissolved entirely when the molten alloy was poured into the form. In the corrosion, copper and tin oxides were noted, while the CuS-inclusions were usually not corroded. The hardness values of 176–245 HV are relatively high and relate both to the high amount of brittle — but hard (α + δ) eutectoid (Mödlinger and Sabatini 2017) — and the high amount of Sn in the alloy. In comparison, hardness measurements in the centre of one of the Cu-drops showed 88 HV. It is also possible that a Sn-rich area beneath the Cu-drop was struck during measuring.

#### Kammerwandgrotte, ID S013, wire

The wire is made of a 12% tin-bronze with about 0.5% S (EDXS), 0.1% Fe, and 0.2% Sb. Other elements, such as Ni, As, and Pb, are present in trace amounts. Silver was not detected, and S and Fe are mainly present in globular CuFeS-inclusions. These inclusions also contain some Pb. Etching the sample with ferric chloride revealed polyhedric grains of different sizes with annealing twins and strain lines. The latter are only present in the smaller grains (Fig. 6d). The wire is porous and cracked in the sampled area, even though it was subjected to light deformation. The slight deformation also resulted in smaller grains close to the surface and bigger ones in the wire’s centre. Corrosion in the sample follows the inter- and intracrystalline structures, especially in the sample’s centre, consisting mainly of copper oxides (cuprite) and carbonates (azurite, malachite). Hardness differs significantly from grain to grain. While the bigger grains in the sample centre are rather soft (81–103 HV), the smaller ones on the edge are much harder (122–170 HV).

#### Prigglitz, inv. no. UF-22692.1780, bracelet

The bracelet is made of tin-bronze with about 12.8% Sn, 0.7% S (EDXS), and 0.1% Fe and Sb. Nickel is present in trace amounts. One end of the bracelet was pinched off, and the cross-section englobed in acrylic resin. Unetched, the sample revealed globular CuFeS-inclusions and (α + δ) eutectoid of the Cu-Sn system that contains up to 0.8% Sb. Etching the sample with ferric chloride and Klemm II revealed coring and 25–40 μm equiaxed grains of α-solid solution with twins. The presence of the (α + δ) eutectoid indicates a low temperature (< 520 °C) or shorter annealing time at higher temperatures. Of particular importance are the drops of pure Cu in the sample surrounded by CuFeS inclusions (Fig. 6e). The drops indicate that not all of the Cu was completely dissolved when the molten alloy was poured into the form. In the final working step, the bracelet was annealed. The corrosion follows both the dendritic structure and grain boundaries and intracrystallinearly between the dislocations (annealing twins) of single grains. As seen under polarized light, and according to the EDXS analyses, both copper carbonates and oxides (shiny, dark-red cuprite crystals) were present, as were various tin oxides. The hardness values of 96–151 HV are in good agreement with an as-cast and annealed 14% tin-bronze.

#### Pottschach, inv. no. 72.488, pin

The pin is made of tin-bronze with about 9.2% Sn, 0.4% S (EDXS), 0.1% Fe, and Sb. Nickel, As, Ag, and Pb are present in trace amounts. The sample was taken from the shaft of the pin, and the cross-section englobed in acrylic resin. The unetched sample revealed CuS-inclusions with a maximum total deformation of about 10%. Porosity in the sample significantly increased towards the centre, corresponding to the centre of the pin shaft. Etching the sample with ferric chloride and Klemm II revealed coring and 25–40 μm equiaxed grains of α-solid solution with twins and strain lines (Fig. 6f). The absence of the (α + δ) eutectoid indicates high temperature or more prolonged annealing. As a final working step, the pin shaft was cold deformed. Corrosion in the sample does not follow the dendritic structure or grain boundaries, or individual grain dislocations (annealing twins and strain lines). Instead, it follows a more irregular pattern typically seen in microbial-induced corrosion (Piccardo et al., 2013). As seen under polarized light, and according to the EDXS analyses, both copper carbonates and oxides (cuprite crystals) are present, as are various tin oxides. Interestingly, the copper oxides form layers on the pin’s shaft surface, while the carbonates are close to the core. The hardness values of 160–193 HV are in good agreement with a cold deformed, previously as-cast and annealed 11% tin-bronze.

## Discussion

All of the axes and chisels in this study are made of tin-bronze with varying amounts of Sn ranging from 7.5 to 10%. They all show coring, indicating that both the duration and temperature of the annealing process were insufficient to homogenize the alloy (Table 3). However, all of the axes and chisels showed equiaxed grains of α-solid solution with twins and strain lines, indicating cold deformation in the final working step due to annealing. CuS-inclusions were found in axes S001 (Prigglitz), S079 (Grünbach am Schneeberg), and the chisel from Kammerwandgrotte, and all other axes contained CuFeS-inclusions. The total amount of deformation for the axes on their edges was astonishingly low overall (usually < 10%), with only axe S001 showing 70–80% of total deformation in combination with (α + δ) eutectoid. The axe also had the highest hardness values of all the objects (300 HV at the edge). Similarly, the chisel showed a significant total amount of deformation of the edge (ca 90%), and in combination with the presence of (α + δ) eutectoid, it had high hardness values (240 HV). The hardness values are directly related to the alloy choice, the total amount of deformation, and especially the deformation applied in the final working step. The influence of the final working step on the end product is demonstrated by the socketed axe from Ternitz (10–20% total deformation) and the chisel (90% total deformation and its eutectoid). Both bronzes contain similar amounts of Sn and Fe, but the socketed axe underwent a higher degree of final deformation than the chisel, which resulted in far higher final hardness.

**Table 3:** Overview of selected metallographic features

No	Site	Object	ID	CuS/Cu_2-x_Fe_2_S	Def. incl.%	Strain lines	Twins	Coring	(α + δ)	Max. HV
1	Prigglitz-Gasteil	Double-pointed ‘awl’	UF-22692.1272	CuS	40–50	x	x	x	-	205
2	Prigglitz-Gasteil	Double-pointed ‘awl’	UF-22692.1140A	CuS	20–30	x	x	x	x	230
3	Prigglitz-Gasteil	Double-pointed ‘awl’	UF-22692.1672	CuS	70–80	x	x	x	-	275
4	Prigglitz-Gasteil, Klausgraben	Axe (socketed)	[S001]	CuS	70–80	x	x	x	x	300
5	Prigglitz-Gasteil	Belt clip	UF-22692.1652	Cu_2-x_Fe_2_S	30–40	-	x	x	-	110
6	Prigglitz-Gasteil	Belt clip	UF-22692.1673	CuS	20–30	-	x	x	-	130
7	Prigglitz-Gasteil	Bracelet	UF-22692.1780	Cu_2-x_Fe_2_S	0	-	x	x	x	150
8	Prigglitz-Gasteil	Casting cake	UF-22692.675	CuS	-	-	-	-	-	105
9	Prigglitz-Gasteil	Knife	UF-10.964	Cu2-xFe2S	globular; edge 60-70	?	x	x	x	255
10	Prigglitz-Gasteil	Knife	UF-22692.2188	Cu2-xFe2S	30–40	x	x	x	-	275
11	Prigglitz-Gasteil	Rod / wand	UF-22692.912	CuS	0	-	-	as-cast	x	245
12	Grünbach, Gelände	Axe (end-winged)	UF-19,452	CuS	30–40	x	x	x	x	220
13	Reichenau, Kammerwandgrotte	Chisel	[S041]	CuS	> 90	x	x	x	x	240
14	Reichenau, Kammerwandgrotte	Wire (bent)	[S013]	Cu2-xFe2S	< 10	-	x	x	-	170
15	Pottschach	Knife	72.485	Cu2-xFe2S	-	-	x	x	-	145
16	Pottschach	Knife	72.484	Cu2-xFe2S	10–20; 30–40	x	x	x	-	230
17	Pottschach	Pin	72.488	CuS	10	x	x	x	-	195
18	Prein an der Rax	Double-pointed ‘awl’	UF-9958	Cu2-xFe2S	0–20	x	x	x	-	180
19	Sieding	Axe (socketed)	UF-5098	Cu2-xFe2S	10–20	x	x	x	-	215
20	Ternitz, Gfieder	Axe (socketed)	[S042]	Cu2-xFe2S	10–20	x	x	x	-	240

Four tin-bronze knives — two from Pottschach and two from Prigglitz — were studied. All four of them contain relatively high amounts of Sn (9 to 15%). Moreover, one of the two Pottschach knives contains 1.2% Sb. All four knives contain S and Fe, which are mainly present in the CuFeS-inclusions. The four knives differ in their post-casting treatment, however. While one knife from Prigglitz (inv. no. 10.964) showed a high amount of total deformation and was cold worked after several annealing/cold deformation steps, which is evident by higher hardness and finer grains, the other (inv. no. UF-22692.2188) showed less total deformation but a higher final cold deformation. The second knife’s treatment resulted in higher hardness values on the edge, even though its tin-amount is much lower (10 versus 15%). Noteworthy, the blade of knife inv.no. 10.964 broke once, was shortened, and attached to a new handle.

The two Pottschach knives show less total deformation than the ones from Prigglitz. Also, knife 72.484 shows slight deformation indications in the final working step and reaches an edge hardness of 230 HV. Knife 72.485 does not show any indications of final deformation; here, annealing was the last working step, resulting in low hardness values of 145 HV or less. The presence of undissolved copper grains in the matrix of knife 72.485 indicates the use of ‘fresh’ copper and tin to produce the alloy or the addition of ‘fresh’ copper to a recycled tin-bronze. All knives show a low amount of total deformation in the core area (max. 20%). While the Pottschach knives and one from Prigglitz (inv. no. UF-22692.2188) are not significantly deformed on the edge, Prigglitz knife 10.964 shows 60–70% of total deformation but less final deformation than the other one from the same site. In combination with the high amounts of Sn and Fe, the result is a relatively high hardness of over 250 HV on the edge. The Prigglitz knife inv. no. UF-22692.2188 shows the knives’ highest hardness value with up to 274 HV (edge) and up to 193 HV (core; ca 2 mm from the edge). The higher HV is due to the final deformation applied in the last working step. Hardness values are higher on the edges of three knives (UF-22692.2188, 10.964, 72.484), while one knife shows a uniform distribution of similar values (knife 72.485). Again, these measurements are in good agreement with the observed microstructures and annealing in the last assumed working step.

Four tin-bronze double-pointed awls — three from Prigglitz and one from Prein — were studied. While two of the awls from Prigglitz are made of tin-bronze with about 10% Sn, the third (inv. no. UF-22692.1672) contains more than 14%. The awl from Prein contains far less at ca 5%. All four awls contain S, which is mainly present in the CuS-inclusions, and Fe was only detected in trace amounts in the awl from Prein. Interestingly, As and Ag were not detected in any of the awls, Ni at trace amounts in two (Prigglitz UF-22692.1672 and Prein) and Sb at about 0.1% in three (Prigglitz UF-22692.1672 and UF-22692.1140A, and Prein). The awl from Prein showed a relatively high amount of Pb at 0.7%, and of the other awls, only the Prigglitz UF-22692.1672 showed traces of Pb. The four awls were studied metallographically and are characterized by very pure Cu in their production. The four awls, however, do not differ in their post-casting treatment but do in its intensity. All but one awl (inv. no. UF-22692.1672) showed heavy coring. While three awls show a total amount of deformation of about 0–50% in the sampled area, only inv. no. UF-22692.1672 showed a much higher level of deformation at 70–80%. All four were cold deformed, and annealed, with cold deformation being the last step of production. In one of the awls (inv. no. UF-22692.1140A), the (α + δ) eutectoid is still present, indicating short, low-temperature annealing, followed by cold deformation. The Prigglitz awl inv. no. UF-22692.1672 shows the highest hardness value of them all at 274 HV. This is due to the intensive total, and especially the final, deformation and the higher amount of Sn in the alloy. The other two awls from Prigglitz had a maximum HV of 230 (inv. no. UF-22692.1140A) and 205 (inv.no. UF-22692.1272). In agreement with the low amount of Sn in the alloy and the low total and final deformation, the Prein awl showed the lowest hardness values between 160–181 HV.

The final annealing of both belt clips makes sense since a final cold deformation would have increased their brittleness. Belt clips need to be flexible. The clips’ production and their hardness values are very similar, as is their chemical composition with the exception of Ag in clip UF-22692.1673, which contains about 0.3% while none was detected in clip UF-22692.1652. Concerning the rod/wand studied, a freshly produced Cu-Sn alloy is presumed, or adding ‘fresh’ Cu to an already existing and recycled tin-bronze, as the microstructure shows not-dissolved Cu-drops, often surrounded by CuS-inclusions. Neither Fe, As, or Ag were detected. The tin-bronze wire was cast and, after some deformation and following annealing, slightly deformed. The bracelet was also cast and slightly deformed and then annealed. Drops of pure Cu indicate that not all of the Cu was dissolved entirely when the molten alloy was poured into the form. The bracelet was annealed for a short time at lower temperatures in the final working step, as (α + δ) eutectoid is still present. The pin was annealed and only slightly deformed after casting. The absence of the (α + δ) eutectoid indicates high temperature or more extended annealing. As a final working step, the pin shaft was cold deformed.

Of particular interest are the undissolved drops of Cu in the CuSn-matrix, which were found in every fifth of the objects (Ternitz, socketed axe (ID S042): twinned grain; Pottschach, knife (72.485): grain; Prigglitz, rod/wand (UF-22692.912): drops with surrounding CuS; and Prigglitz, bracelet (UF-22692.1780): big drops). The drops range from 5 to 35 μm in diameter. Such unalloyed copper inclusions (UCI) have been noted in other archaeological bronzes (Bosi et al. 2002). The UCI observed in the four objects discussed here do not relate to corrosion, which are usually irregularly shaped from pseudo-morphically replacing other phases and are instead due to the (s)melting process itself. For the objects in this study, not all of the Cu was completely dissolved when the molten alloy was poured into the casting mould.

The CuS-layers around the copper drops of the rod/wand (UF-22692.912) suggest the copper drops might have already formed during the smelting process (Bosi et al. 2002). Above 1105 °C, Cu and S (< 20%) are immiscible with a top layer of Cu_2_S and a bottom layer of Cu with about 2% S (copper-rich Cu–S solution). Upon cooling, the solid phase Cu_2_S and the copper-rich Cu–S solution are present, and slagging of solid Cu_2_S takes place to obtain high-purity copper. During cooling, spherical particles of Cu_2_S containing a copper-rich core might form, which are heavier than Cu_2_S particles and therefore less likely to slag and remaining in the alloy to form UCI (Bosi et al. 2002). While singular copper drops in crystallized form were noted for socketed axe (ID S042) and the knife (72.485), many of them with up to 35 μm diameter, and most had already oxidized to CuO, were noted on bracelet (UF-22692.1780). For these three objects, we can either assume that fresh copper was added to an already existing CuSn-alloy (recycling) or that the CuSn alloy was freshly produced. Hence, we can assume that objects were very much likely not made using recycled tin-bronzes.

Regarding the objects’ function, all cutting edge objects were worked to improve their material properties, especially their edge hardness. Hardness values differed for the jewellery, except for the pin where the last working step was annealing, and hardness values are thus comparatively low at between 110 and 170 HV. These values suggest that elasticity was more important than hardness. Only the as-cast rod/wand from Prigglitz shows relatively higher hardness values, which are related to the presence of the eutectoid.

Concerning cutting edge objects (axes, chisels, and pickaxe), hardness values range from 215 to 300 HV, highlighting that the material properties of the edges were intentionally improved for an ideal usability of the tools. The only exception is knife 72.485 from Pottschach with 145 HV.

Hardness values depend on various factors such as alloy composition, thermal treatment, and deformation applied. For the objects studied, this is particularly the Sn and Fe content. The presence of Fe, even at values as low as 0.15%, also reduces formability and causes cracks and surface defects (see Nerantzis 2015; Papadimitriou 2008). It also influences the eutectoid and, most importantly, the final deformation treatment that was applied. As no direct correlation between Fe or Sn content, final deformation, and hardness values were detected (likely also related to the low number of samples), we assume that Bronze Age smiths simply stopped at a certain point during the final working step (cold deformation) once they reached a (for them) sufficiently good result. This does not necessarily need to be the highest possible hardness of the alloy, as was also observed often at Early Bronze Age axes from north Alpine regions (Kienlin 2008).

## Conclusions

The Late Bronze Age site of Prigglitz-Gasteil is considered a regional centre of copper production and bronze working based on the evidence of an openwork copper ore mine and a nearby bronze casting workshop. Metallographic analyses of different cutting tools and jewellery items from Prigglitz-Gasteil, and six contemporaneous sites in the surrounding region, provide insights into the post-casting treatment of different kinds of bronze objects. This information helps us understand the overall *chaîne opératoire* in metal production, which is important in identifying local metalworking traditions.

As discussed in detail, objects with a cutting edge were worked according to their function. For instance, the sharp edges of axes and knives and the points of the awls had undergone annealing and cold deformation cycles with cold deformation as the final step in almost all cases. This sequence of treatments resulted in higher hardness. The total deformation on blade edges ranged from 10 to 20% (one awl, two socketed axes), 30–50% (end-winged axe, two awls, two knives), 60–70% (one knife, one awl, one socketed axe), and beyond (chisel with over 90%). Interestingly, these objects’ alloys do not seem to have been chosen intentionally to include higher Sn concentrations except for awl UF-22692.1672 and knife 10.964 from Prigglitz.

Small jewellery objects, such as the belt clips, the bracelet, and wire, which need to have a certain amount of flexibility to function, were worked far less with a total amount of deformation of 0–40%. Their last working step was annealing, which resulted in higher flexibility and lower hardness. Comparatively, the pin from Pottschach received a final cold deformation treatment, resulting in higher hardness. Also of note, the hardness of the as-cast 12% tin-bronze rod/wand fragment from Prigglitz is due to the presence of (α + δ) eutectic with 245 HV.

There was no distinct difference in quantity and quality of post-casting treatments for the objects from the sites surrounding the Prigglitz-Gasteil mine. A pattern that seems to arise, albeit with the limited number of samples investigated, suggests that the Prigglitz region’s bronze production was not standardized. Alloys do not seem to have been explicitly chosen for particular object types or their intended function; however, this conclusion should be revisited as additional samples are investigated in the future. Finally, it is worth mentioning that four out of the 20 studied objects (Ternitz, socketed axe; Pottschach, knife; and Prigglitz, rod/wand and bracelet) contain unalloyed copper inclusions, which are most likely related to the incomplete mixing of scrap metals and alloys during recycling.
